# Factors and pathways of non-suicidal self-injury in children: insights from computational causal analysis

**DOI:** 10.3389/fpubh.2024.1305746

**Published:** 2024-03-12

**Authors:** Xinyu Guo, Linna Wang, Zhenchao Li, Ziliang Feng, Li Lu, Lihua Jiang, Li Zhao

**Affiliations:** ^1^Department of Health Policy and Management, West China School of Public Health and West China Fourth Hospital, Sichuan University, Chengdu, China; ^2^College of Computer Science, Sichuan University, Chengdu, Sichuan, China; ^3^Teaching and Research Section of General Practice, The General Practice Medical Center, West China Hospital of Sichuan University, Chengdu, China

**Keywords:** NSSI, causal discovery, mental health, artificial intelligence, risk factors, COVID-19

## Abstract

**Background:**

Non-suicidal self-injury (NSSI) has become a significant public health issue, especially prevalent among adolescents. The complexity and multifactorial nature of NSSI necessitate a comprehensive understanding of its underlying causal factors. This research leverages the causal discovery methodology to explore these causal associations in children.

**Methods:**

An observational dataset was scrutinized using the causal discovery method, particularly employing the constraint-based approach. By integrating machine learning and causal inference techniques, the study aimed to determine direct causal relationships associated with NSSI. The robustness of the causal relationships was evaluated using three methods to construct and validate it: the PC (Peter and Clark) method, Fast Causal Inference (FCI) method, and the GAE (Graphical Autoencoder) method.

**Results:**

Analysis identified nine nodes with direct causal relationships to NSSI, including life satisfaction, depression, family dysfunction, sugary beverage consumption, PYD (positive youth development), internet addiction, COVID-19 related PTSD, academic anxiety, and sleep duration. Four principal causal pathways were identified, highlighting the roles of lockdown-induced lifestyle changes, screen time, positive adolescent development, and family dynamics in influencing NSSI risk.

**Conclusions:**

An in-depth analysis of the factors leading to Non-Suicidal Self-Injury (NSSI), highlighting the intricate connections among individual, family, and pandemic-related influences. The results, derived from computational causal analysis, underscore the critical need for targeted interventions that tackle these diverse causative factors.

## 1 Introduction

Non-suicidal self-injury (NSSI) has become a pressing public health issue, with rising prevalence both in developed and developing countries (1). Rates of NSSI fluctuate between 11.5 and 33.8%, contingent on sample type and study design, but there is an undeniable uptrend worldwide, even in developing nations. Adolescence is the peak risk period for NSSI (2), with about 23% of adolescents, 13.4% of young adults, and 5.5% of adults being affected (3). Alarmingly, up to 24.7% of Chinese adolescents report experiencing NSSI (4), which necessitates further attention (5).

The repercussions of NSSI in children and adolescents are severe and long-lasting (6, 7). It is closely linked to heightened suicidal ideation and attempts (8). Even when accounting for suicidal thoughts, NSSI remains a potent predictor of suicidal actions (9–11). Specifically, 39.6% of those who have self-harmed report suicidal behaviors, and of that cohort, 66.3% have a history of NSSI (12). Besides, NSSI correlates with several psychological challenges, like depression, anxiety, and post-traumatic stress disorder(PTSD), and negatively impacts familial and interpersonal bonds (13, 14).

The risk factors for non-suicidal self-injury in children and adolescents are diverse and multifaceted. While much emphasis has been placed on the psychological, family, and school levels—including psychological disorders and symptoms, adverse family experiences, and victimization (4, 15, 16)—the underlying mechanisms driving NSSI behaviors are complex. Many studies to date have been constrained by methodological limitations, failing to grasp the problem from a comprehensive and systematic viewpoint. Moreover, the emergence of the COVID-19 pandemic and its associated lockdown measures has further complicated the scenario (17, 18). Factors such as changes in sleep time (19), physical activity, screen time (20), and increased stress due to isolation and academic pressures have heightened the mental and emotional issues among children and adolescents, possibly leading to a surge in NSSI behaviors (21–23).

Protective factors against Non-Suicidal Self-Injury (NSSI), including life satisfaction and Positive Youth Development (PYD), have been highlighted as promising avenues for intervention. Research highlights that higher levels of life satisfaction can serve as a significant buffer against self-injurious behaviors, while the presence of PYD qualities has been shown to not only reduce the risk of NSSI but also lessen the impact of depressive symptoms on such behaviors (24–26). This underscores the PYD perspective's shift from focusing on youth deficits or psychopathology to emphasizing their strengths, skills, and assets, which can be nurtured and improved (27, 28). These factors are theorized to buffer individuals from the deleterious effects of risk factors like depression and family dysfunction. Enhancing these protective mechanisms may mitigate the risk of NSSI, providing a critical strategy for supporting at-risk adolescents.

**Figure 1:**
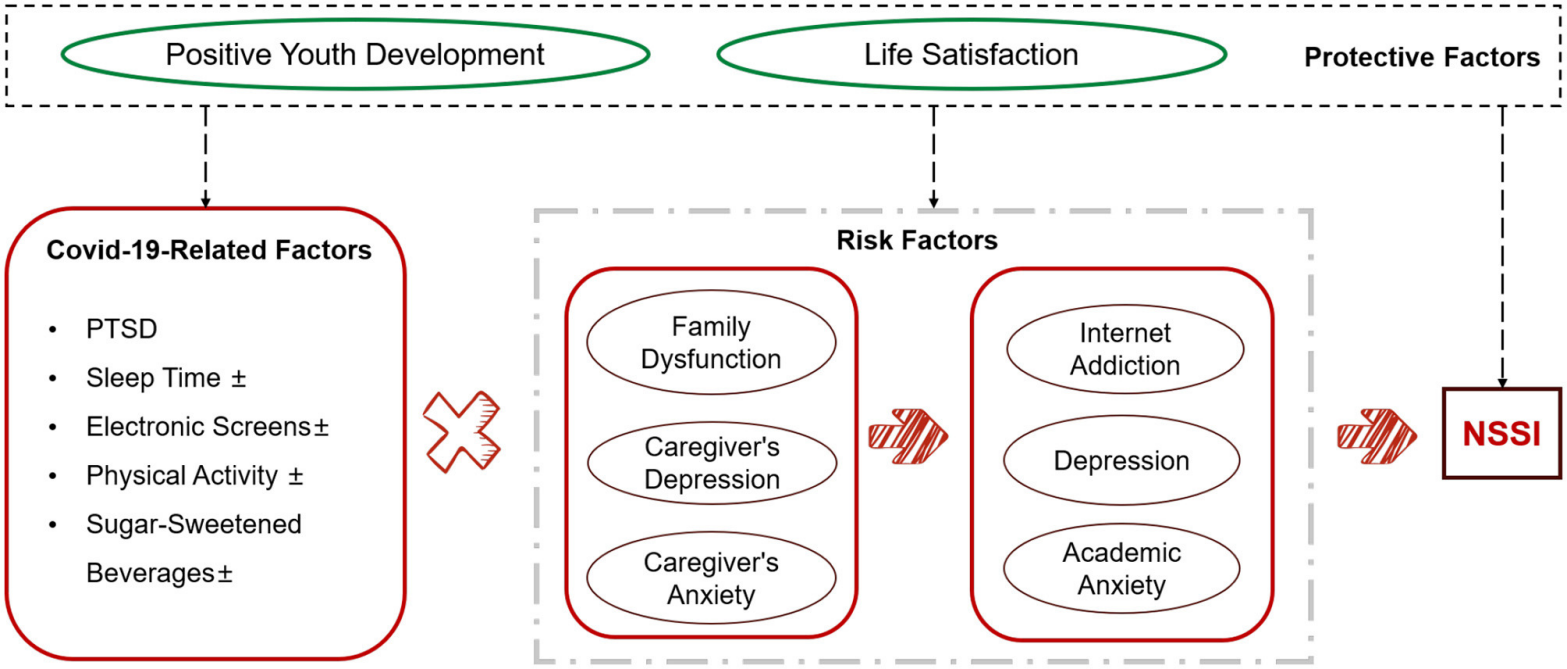
NSSI comprehensive hypothetical framework.

Based on the literature review above and the four-function model of self-injury (29, 30), we developed a comprehensive hypothetical framework (Figure 1) for the emergence of NSSI in adolescents. we have formulated a series of causal pathway hypotheses to be explored within our comprehensive hypothetical framework.

- Changes in sleep times, physical activity, consumption of sugar-sweetened beverages, and screen time during COVID-19 lockdowns are hypothesized to augment the probability of developing familial and psychological dysfunctions, subsequently enhancing the risk of NSSI.
- COVID-19-related PTSD is presumed to escalate the risk of psychological issues, further contributing to NSSI.
- Family dysfunction may indirectly lead to NSSI by exacerbating psychological distress.
- PYD qualities could potentially modulate the relationship between psychological distress and NSSI.

Building on the comprehensive review and the hypothetical framework outlined above, this research adopts causal discovery methods within a robust theoretical framework to elucidate the complex web of factors influencing NSSI among adolescents. Traditional research methodologies, which are predominantly observational and correlational, have contributed valuable insights yet frequently encounter limitations in establishing causality (31). Moreover, there is a tendency within existing research to adopt a narrow focus on isolated variables, neglecting the broader constellation of contributing factors. Recognizing the limitations of conventional research methodologies in capturing the multifaceted nature of NSSI, our study utilizes a causal discovery approach applied to a unique observational dataset, aiming to identify causal factors associated with NSSI in children.

Causal discovery, a methodological paradigm dedicated to unearthing cause-and-effect relationships among variables, emerges as a formidable tool in scientific inquiry and data analysis. It enables researchers to transcend the boundaries of correlation, probing the underlying “why” behind observed phenomena (32–34). In the domain of NSSI, discerning the causal dynamics behind this complex behavior is crucial for crafting effective prevention and intervention measures. Through this approach, our study aims to contribute a nuanced understanding of NSSI, fostering the development of more precise and efficacious strategies to combat this pressing issue among youth.

## 2 Materials and methods

### 2.1 Study design and participants

Data for this research was sourced from the Chengdu Positive Child Development (CPCD) survey (35). Launched in December 2019 in Chengdu, this school-based longitudinal study targeted students aged 6–16 years, drawn from five primary and secondary schools. Information was collected using a mix of paper and electronic questionnaires completed by children and caregivers, as well as through direct interviews. The study aimed to explore connections between students' sociodemographic factors, health, lifestyle behaviors, and academic performance, with supplementary data obtained from caregiver health evaluations and school records. The first follow-up of the survey, conducted between June 16, 2020, and July 8, 2020, aligned with the resumption of classroom activities following the COVID-19 lockdowns.

**Figure 2:**
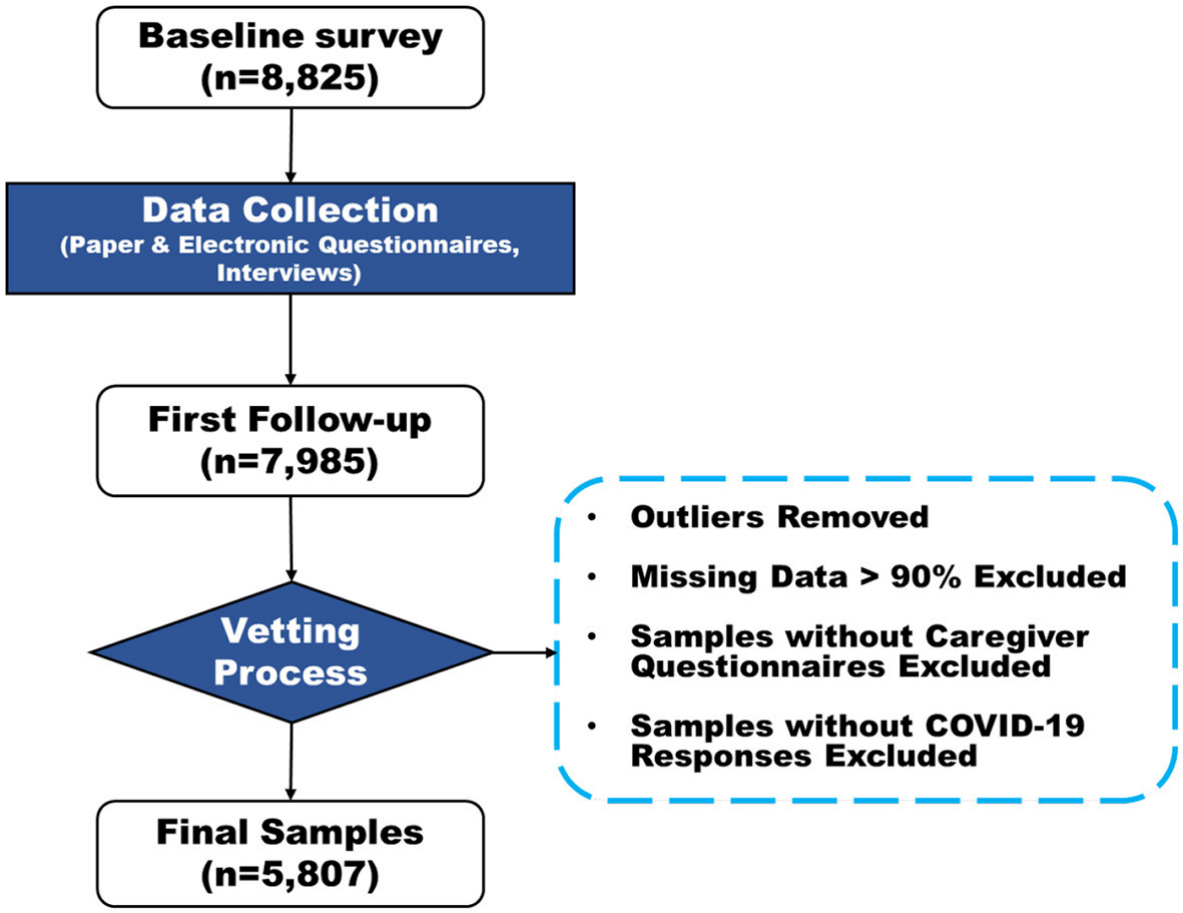
Sample selection and refinement process for NSSI study.

A meticulous vetting process was utilized to refine the dataset, ultimately including 5,807 students to ensure high-quality data for robust causal inference. This process involved identifying and eliminating statistical outliers, removing variables with a missing rate exceeding 90%, and excluding samples lacking caregiver questionnaires or COVID-19 related responses. The “Sample Selection and Refinement Process for NSSI Study” is illustrated in Figure 2.

### 2.2 Ethics statement

The confidentiality of all data was meticulously maintained by the research team, with no disclosure of any personal information within the study's findings. This research adhered to the principles set forth in the Helsinki Declaration, secured approval from the Ethics Committee of Sichuan University (Approval No. K2020025), and obtained consent from the relevant school authorities, parents, and students.

### 2.3 Measures

#### 2.3.1 Non-suicidal self-injury

The assessment of Non-suicidal self-injury (NSSI) in our study was conducted using the 9-item Intentional Self-Injury Scale, developed and validated by Gratz (36). This scale evaluates eight specific NSSI behaviors, including cutting, burning, biting, stabbing, hitting, pinching, and ingesting non-food items, along with a self-assessment for NSSI-related hospitalizations. Each behavior is rated on a 4-point scale, ranging from 1 (never) to 4 (three or more times). Participants reporting a score of 1 or higher on any item were identified as exhibiting NSSI behaviors. This measurement approach has been previously validated in studies involving Chinese adolescents (37).

#### 2.3.2 Explanatory variables

In our research, we carefully selected 21 variables collected during the first follow-up period of the CPCD Survey. This selection was strategically guided by the existing literature (4, 8–10, 15, 38), and the aim of our study was to explore causal relationships with non-suicidal self-injury (NSSI). We focus on various sociodemographic, behavioral, and psychological factors that have been previously associated with NSSI behaviors. This approach aimed to create a manageable and comprehensive analytical framework that aligned our study with established findings and theory in NSSI research.

Sociodemographic data, including gender, age, grade, and BMI, were collected, prioritizing “Grade” for its relevance to educational context and peer interactions. Mental health status and psychological characteristics were evaluated through scales measuring depression, academic anxiety, positive child development, life satisfaction, family dysfunction, and internet addiction, aiming to create a comprehensive analytical framework that aligns with established NSSI research findings and theories.

Moreover, the study evaluated COVID-19-related behavioral factors like PTSD, changes in sleep patterns, physical activity, screen time, and sugary beverage consumption to understand the pandemic's impact on students' routines and its potential link to NSSI behaviors. These evaluations used precise binary coding for behavioral changes, offering detailed insights into lifestyle adjustments during the pandemic. Additional considerations included internet addiction and caregivers' mental health status, with a comprehensive overview of all variables, definitions, and measurement methods provided in the Supplementary material.

### 2.4 Data pre-processing

We conducted essential data preprocessing steps to ensure that we had a well-prepared dataset for subsequent causal discovery analysis. To harmonize the numerical and categorical features within the dataset, we employed standardized procedures. For the categorical variables, which included ordinal data with a discernible degree of ordering, we applied the LabelEncoder technique to transform them into numerical representations while preserving the ordinal relationships. Simultaneously, we standardized the numerical features using the StandardScaler method, which centered the variables around a mean of zero and scaled them to unit variance. This preprocessing not only alleviated potential issues arising from varying scales but also facilitated the compatibility of our data with the causal discovery algorithm, ensuring that it operated effectively in uncovering causal relationships within our dataset.

### 2.5 Causal discovery model application

It is crucial to recognize that causality and correlation are distinct concepts with profound implications for scientific research and data analysis. While correlation measures the strength and direction of statistical association between variables, it does not elucidate the direction of influence or establish a cause-and-effect relationship. Causality, on the other hand, delves into the intricate web of cause-and-effect connections, emphasizing that changes in one variable lead to changes in another. In this study, we aimed to identify the complex causality hidden in NSSI based on social-demographic characteristics, psychological characteristics, COVID-related behavioral factors, behavioral factors, mental health status and caregiver's mental health status.

The primary methods for learning the causal graph structure among variables include constraint-based, score-based, and gradient-based approaches. Constraint-based methods, such as PC (Peter and Clark) and FCI (39), identify causal relationships by recognizing patterns of conditional independence. Score-based methods, including GES (40), assign numerical scores to different causal structures and search for the structure with the highest score. Gradient-based methods, including GAE (41), have emerged as notable alternatives, exhibiting superior accuracy and computational efficiency. In comparison to constraint-based and score-based methods, gradient-based methods directly learn the causal graph through end-to-end optimization, eliminating the need for explicit detection of conditional independence or the use of scoring functions in intermediate steps.

**Figure 3:**
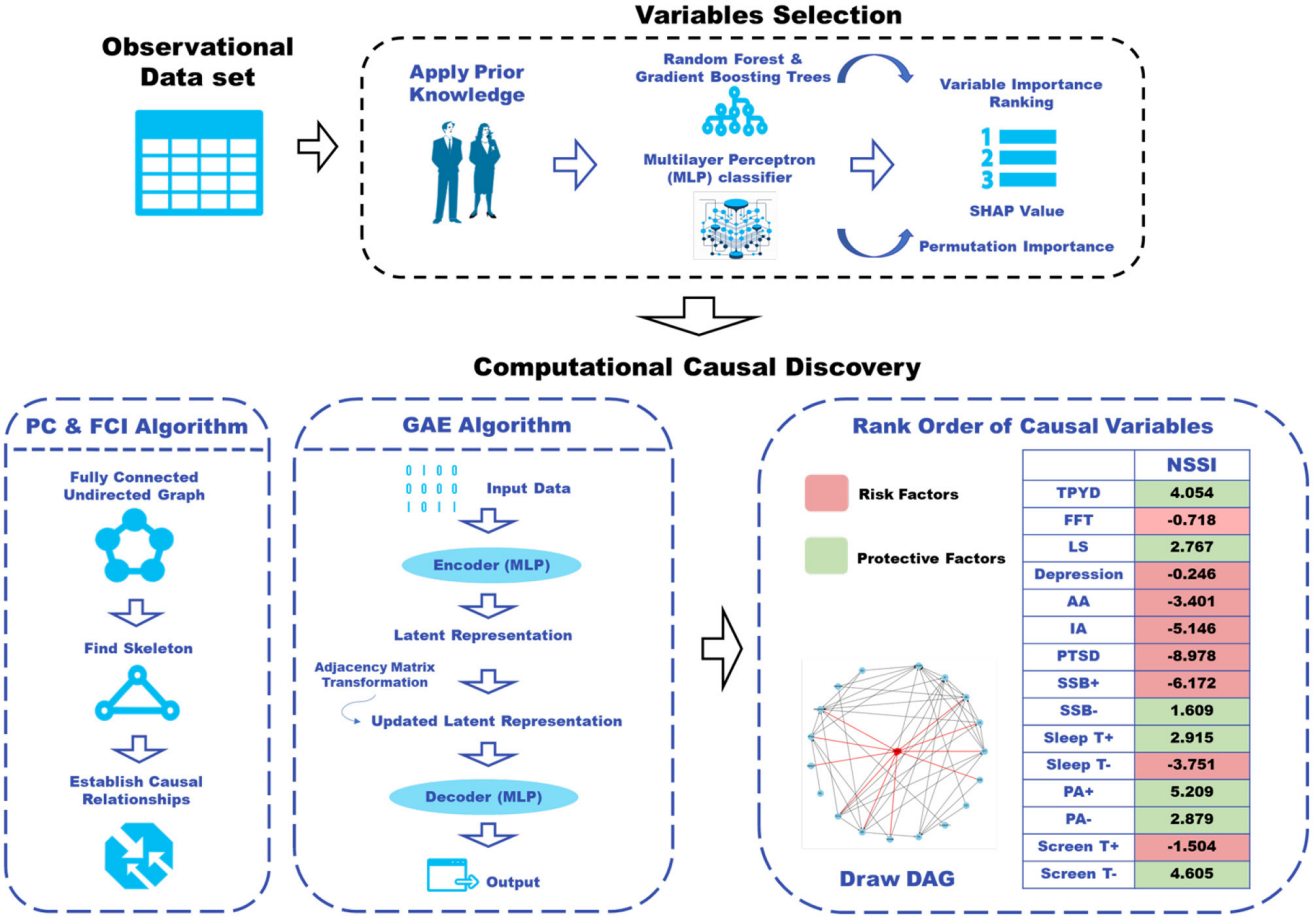
The workflow for computational casual discovery.

Consequently, having recognized the strengths of each approach, we integrate a combination of these methods to enhance the accuracy and robustness of our causal Directed Acyclic Graph (DAG) learning. We employed three approaches for constructing and validating the DAG: the PC method (34), FCI (42), and the GAE. This is aimed at fortifying the reliability and credibility of our DAG, ensuring its resilience and accuracy across diverse analytical frameworks. The framework of our prediction work is presented in Figure 3.

**PC algorithm** represents a fundamental approach to causality analysis that is grounded in conditional independence testing. Its fundamental logic hinges upon the principle that genuine causal relationships manifest as conditional independence relationships in observational data. **FCI algorithm** is an extension of PC, designed to handle latent variables more effectively. As both PC and FCI are Constraint-based methods, they first use conditional independence tests to learn the skeleton of the underlying casual graph, and then orient the edges based on a series of orientation rules. It's important to note that the order in which variables are considered can influence the outcomes of these two algorithms.

To mitigate the impact of order-dependency, we conducted multiple runs of both PC and FCI with different random orders of variables. This ensures that the algorithm are exposed to various variable orders, mitigating the influence of a specific sequence on the final results. In our study, for FCI and the three variations of PC (original, stable, and parallel), we performed 1,000 runs for each, employing random variable orders. We identified the edges that appear most frequently across the multiple runs. The final structure, represented by edges that consistently appear, can be considered as the more robust DAG. The general steps for PC and FCI are described as follows:

1. Prior knowledge on variable selection. It is crucial to note that the PC and FCI algorithm assume causal sufficiency, and therefore, all potential confounding factors should be present in the data for a comprehensive causal analysis. We conscientiously leveraged prior knowledge in the face of a large number of total features to identify the variables relevant to NSSI.

2. Prior knowledge on variable relationship. We added prior knowledge to guide our causal modeling by informing the anticipated forbidden causal pathways. The rationale behind the prohibition of each path is detailed below.

- Prohibition of paths leading to “Grade” and “Gender” variables. Within our causal modeling framework, we have enforced a stringent constraint by prohibiting any causal pathways that lead to the “Grade” and “Gender” variables. This restriction is grounded in the fundamental premise that “Grade” and “Gender” are considered intrinsic attributes of individuals, impervious to external causal influences.
- Prohibition of paths leading to “COVID-Related Behavioral Factors”. We also prohibited any causal pathways leading to the “COVID-Related Behavioral Factors”. This constraint arose from the distinctive temporal characteristics and underlying nature of these factors. Unlike other variables in our analysis, which were sourced from a single survey conducted during the COVID-19 pandemic, the “COVID-Related Behavioral Factors” represented the dynamic changes observed between this COVID-19 period survey and another before-COVID-19 period survey. The timeline of these behavioral factors is inherently distinct, encompassing the evolving responses of individuals to the unique circumstances brought about by the pandemic. Thus, we enforced the prohibition of all paths leading to “COVID-Related Behavioral Factors”.

3. Causal discovery process. Our application of the PC and FCI algorithms followed these essential steps:

- Skeleton discovery: Begin with a fully connected undirected graph that includes all variables. Utilized conditional independence tests to uncover the initial skeleton of the causal graph, representing potential pairwise relationships between variables. We performed independence test using Fisher-*z*'s test.
- Edge Orientation: PC orients edges based on conditional independence tests. FCI orients edges within Markov equivalence classes, considering latent variables and capturing more complex causal relationships.
- Refinement: PC refines the graph iteratively by applying conditional independence tests and edge removals. FCI refines the graph by considering additional conditional independence tests and making decisions about edge orientations within Markov equivalence classes.
- Multiple runs and final structure: For FCI and three variations of PC, we performed 1,000 runs with random variable orders. Identified edges that consistently appeared across multiple runs to establish a final structure.

**GAE algorithm** represents Graph Autoencoder for causal structure learning. It is an alternative generalization of NOTEARS (43) to handle nonlinear causal relations. After decoder, GAE can generate an adjacency matrix that captures the relationships between nodes. The adjacency matrix typically includes positive values for positive edges and negative values for negative edges. Positive value might represent a positive relationship or interaction, while negative value might represent a negative or inhibitory relationship. We used the learned latent representations from the GAE to identify factors associated with protective effects and those contributing to an increased risk, shown in Figure 3.

## 3 Results

### 3.1 Sample characteristics

Among the 5,307 participants included in the final analysis, 1,394 individuals (26.27%) reported engaging in non-suicidal self-injury (NSSI) in the past year. Among the 1,394 participants who reported engaging in NSSI, the NSSI detection rate was 24.82% for males (*n* = 667) and 27.75% for females (*n* = 727). The NSSI detection rate among primary school students was 23.51% (*n* = 886), while among middle school students, it was 33% (*n* = 508). The characteristics of the sample are presented in Table 1.

**Table 1:** Sample characteristics.

	**Total (*n* = 5,307)**	**NSSI (*n* = 1,394)**	**Incidence of NSSI (%)**
**Gender**			
Male	2,687 (50.63)	667 (47.85)	24.82
Female	2,620 (49.37)	727 (52.15)	27.75
**Grade**			
1	239 (4.50)	33 (2.37)	13.81
2	312 (5.88)	49 (3.52)	15.71
3	852 (16.05)	200 (14.35)	23.47
4	1,019 (19.20)	238 (17.07)	23.36
5	664 (12.51)	188 (13.49)	28.31
6	682 (12.85)	178 (12.77)	26.10
7	807 (15.21)	247 (17.72)	30.61
8	732 (13.79)	261 (18.72)	35.66
Primary school	3,768 (71.00)	886 (63.56)	23.51
Junior high school	1,539 (29.00)	508 (36.44)	33.01
**BMI**			
Normal	4,169 (78.56)	1,103 (79.12)	26.46
Overweight	454 (8.55)	126 (9.04)	27.75
Obesity	684 (12.89)	165 (11.84)	24.12
**COVID-19-related behavioral factors**			
Sleep time+	1,401 (26.40)	363 (26.04)	25.91
Sleep time−	2,470 (46.54)	714 (51.22)	28.91
Physical activity+	1,796 (33.84)	470 (33.72)	26.17
Physical activity−	2,233 (42.08)	618 (44.33)	27.68
Screen time+	1,387 (26.14)	398 (28.55)	28.70
Screen time−	2,521 (47.50)	692 (49.64)	27.45
SSB+	727 (13.70)	247 (17.72)	33.98
SSB−	1,963 (36.99)	570 (40.89)	29.04
**Mental health status**			
No depression	3,449 (64.99)	507 (36.37)	14.70
Depression	1,858 (35.01)	887 (63.63)	47.74
No anxiety	4,472 (84.27)	896 (64.28)	20.04
Anxiety	835 (15.73)	498 (35.72)	59.64
Academic anxiety (< 4)	3,366 (63.43)	787 (56.46)	23.38
Academic anxiety (≥4)	1,941 (36.57)	607 (43.54)	31.27
No PTSD	3,468 (65.35)	710 (50.93)	20.47
PTSD	1,839 (34.65)	684 (49.07)	37.19
**Internet addiction**			
Average	4,514 (85.06)	987 (70.8)	21.87
Occasional to frequent	722 (13.60)	363 (26.04)	50.28
Significant	71 (1.34)	44 (3.16)	61.97

### 3.2 Direct causes and effects of NSSI (local causal network)

We utilize a directed acyclic graph (DAG) to exhibit the complexity of variables influencing adolescent and child non-suicidal self-injury (NSSI) behaviors during the COVID-19 pandemic. Within this illustrative graph, the structure of the local causal network model, fundamental to our study, is evident. It includes an array of variables, each having its significance in the broader scope. The DAG aptly captures the “upstream” and “downstream” variables (31). Here, “upstream” variables act as forerunners or primary triggers that might affect succeeding variables. Conversely, the “downstream” variables are those likely influenced or modified due to the preceding ones.

**Figure 4:**
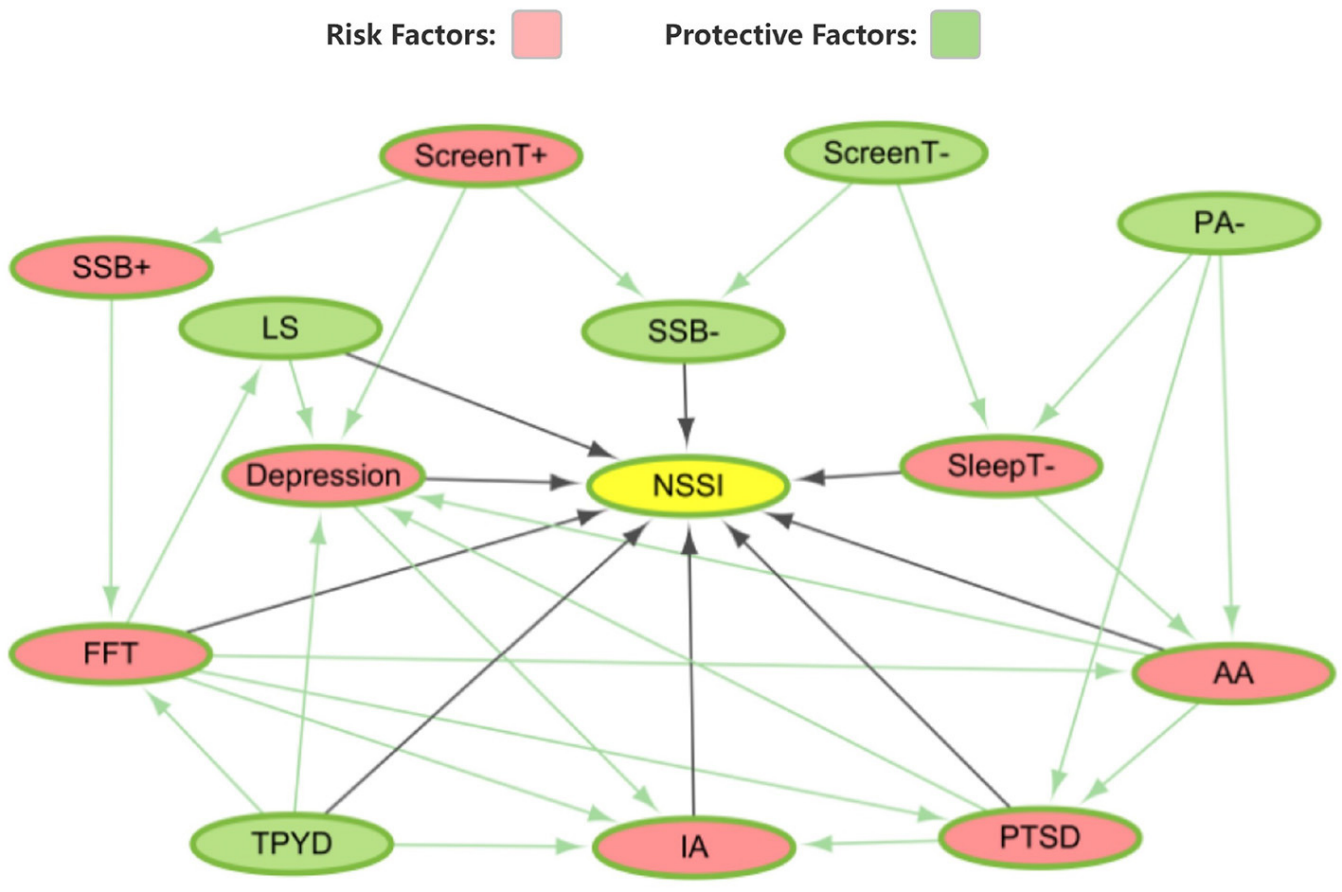
The DAG for local variable in NSSI.

The DAG offers a comprehensive visualization, revealing both direct neighbors and secondary neighbors within two “steps” of NSSI behaviors. This graphical representation paints an in-depth portrait of the relationships between various factors during the COVID-19 pandemic's acute phase for adolescents. Our application of the GAE algorithm not only corroborates these findings but also enriches them by providing a nuanced understanding of the factors contributing to NSSI. This advanced method, represented in Figure 3, facilitated the quantification of each variable's impact by calculating their polarity and magnitude. The GAE algorithm's unique ability to model nonlinear relationships allowed us to further delineate factors with protective effects (denoted in green) from those associated with increased risk (indicated in red). The results, as depicted in Figure 4, confirm and strengthen our causal network model, providing a nuanced understanding of the variables that directly or indirectly contribute to NSSI behaviors.

The nodes with direct causal relationship with NSSI are life satisfaction, depression, family dysfunction, sugary beverage consumption, PYD, Internet addiction, COVID-19 related PTSD, academic anxiety and sleep duration. The analysis identified four key causal pathways influencing NSSI: (1) Lockdown-induced reductions in physical activity escalate academic anxiety, potentially leading to PTSD, heightening internet addiction and NSSI risks. (2) Enhanced screen time and sugary beverage consumption are linked to elevated depression risks and increased NSSI likelihood. (3) Positive adolescent development acts as a buffer, mitigating the adverse effects of family dysfunction and internet addiction on NSSI. (4) Family dysfunction negatively impacts life satisfaction and fosters depression, directly contributing to NSSI. The detailed DAG illustrating these relationships is provided in Supplementary Figure S1.

The graph further enlightens us about individual attributes that could influence this dynamic, underscoring the phenomena's multifaceted nature. The focus on NSSI behavior in the DAG, and its potential links with these various factors, is particularly noteworthy. Moreover, the graph showcases the intricate ties between these variables, elucidating the ripple effects and possible feedback mechanisms within the system. Ultimately, the DAG serves as a comprehensive visual tool, elucidating the intricate connections and potential causal pathways pertaining to NSSI behavior in adolescents amid the COVID-19 pandemic.

## 4 Discussion

Our research harnessed machine learning and causal discovery techniques to understand the intricate web of causal links surrounding NSSI in children and adolescents. Aiming to shed light on influential factors related to individual characteristics, family dynamics, and the repercussions of the COVID-19 pandemic, we strived to provide actionable insights that could spearhead innovative prevention and intervention approaches. Given the complex nature of NSSI, dictated by numerous variables, and the limitations of current research methodologies, we sought a fresh approach to discern causation, ultimately guiding more effective strategies. The PC and FCI algorithms exhibit sensitivity to the order of variables, implying that the obtained results may vary based on the sequence in which variables are considered during execution. To address this, we conducted multiple runs of these algorithms using different orders of features. Additionally, to enhance the robustness of the causal structure, we incorporated the GAE method into our approach. Subsequently, the most frequently identified edges were retained for further in-depth analysis.

Our study has delineated a network of nine pivotal variables that exhibit direct causal associations with NSSI behavior in children, as visualized in Figure 4 (for the full names and assignments corresponding to the abbreviations of the variables shown in Supplementary Table S1). Central among these findings is the pervasive influence of the COVID-19 pandemic, which manifest as a marked decrease in sugary beverage intake, diminished sleep quality, and the emergence of COVID-19-triggered PTSD. Individual psychological drivers encompassed themes of academic stress and manifestations of depression. In tandem, key psychological attributes were identified: the holistic life satisfaction measure, the nuanced interplays of family dynamics, the embodiment of Positive Youth Development (PYD) (26), and the grip of internet addiction. The coherence of these findings with existing literature underscores the robustness of our methodological approach (4, 44, 45).

Significantly, the COVID-19 pandemic has altered the sleep patterns of children and adolescents, a crucial aspect of mental health. Prolonged lockdowns have disrupted regular sleep schedules, heightening stress and anxiety levels. These sleep disruptions, combined with the stress of new learning modes and social isolation, have intensified academic anxieties, affecting family dynamics and increasing household tensions. Moreover, the pandemic has triggered PTSD in young people, a concerning development given its long-term mental health implications. This emergence of PTSD, fueled by constant pandemic-related news and personal experiences, adds a critical dimension to our understanding of the pandemic's impact on youth mental health.

Digging deeper into these variables, the interconnectedness within this network is profound. Academic stress finds its roots in the diminished sleep and curtailed physical activity patterns during the pandemic, as well as the challenges posed by family dynamics. The haunting presence of COVID-19-related PTSD is sculpted by the restrictive physical activity regimes, academic stressors, and family dysfunction. Depression's emergence is further amplified by excessive screen time, both for academic and recreational purposes, interwoven with academic anxieties, the nurturing aspects of Positive Youth Development, and overarching life satisfaction. Completing this intricate web, internet addiction bears the imprints of PTSD linked to the pandemic, the shadows of depression, family struggles, and the protective or exacerbating elements of Positive Youth Development.

Emerging from our findings is a comprehensive understanding that provides invaluable insights for safeguarding the mental wellbeing of children and adolescents in the wake of unforeseen public health crises, such as the COVID-19 pandemic. This understanding emphasizes the potential of our data in guiding practical interventions and preventive measures. The notable reduction in sugary beverage consumption and sleep duration, as well as the emergence of pandemic-induced PTSD, highlight the profound physiological and psychological shifts induced by prolonged lockdowns and associated societal changes (46, 47). Our findings advocate for prevention strategies that are not only trauma-informed but also adaptive to the evolving public health landscape.

The established causal relationship between sugary beverage consumption and NSSI echoes previous studies associating unhealthy diets with increased depressive symptoms in adolescents (48, 49). Inflammatory diets can intensify mental health problems, possibly through obesity and inflammation (50, 51). Thus, the importance of balanced diets is underscored. Emphasizing a diet rich in fruits, vegetables, and anti-inflammatory foods, combined with strategies like restoring regular sleep patterns and trauma-informed interventions, may offer a comprehensive approach to improving the mental wellbeing of children and adolescents.

Furthermore, understanding the intricate network of causal factors—including academic anxieties (52), family dynamics (53), and individual psychological attributes—means that interventions can be more targeted and precise. This precision is vital in the clinical setting, where tailored interventions can lead to more effective outcomes. Rather than applying generic measures, strategies can be devised to specifically counteract or augment identified causal agents. This approach enhances the practical utility of our findings, offering a roadmap for clinicians and policymakers in developing targeted interventions. The identified drivers, such as academic stress and depression, could be targeted through school-based programs emphasizing coping mechanisms, emotional regulation, and peer support. Family-centered interventions might focus on strengthening familial bonds and improving communication, reducing the chance of family dysfunction exacerbating mental health issues (54). The correlation between internet addiction and NSSI emphasizes the importance of digital literacy programs that equip adolescents with skills to navigate the online world safely.

A key distinction of our study lies in the use of computational causal discovery. Traditional methodologies often restrict themselves to observational correlations, which, although informative, don't offer a genuine window into the underlying causative structures. Causal discovery goes beyond merely identifying these associations, allowing us to pinpoint the drivers of adverse outcomes such as NSSI. The superiority of this approach lies in its potential to tailor prevention and intervention strategies based on the actual causes, rather than mere symptoms or correlated factors. This means that initiatives informed by our findings can be significantly more effective, as they strike at the heart of the issue, directly addressing and mitigating the root causes. As we move forward in our collective endeavor to nurture the mental health of our younger generation, leveraging advanced methodologies like computational causal discovery will be paramount in ensuring our strategies are not only well-informed but also impactful.

## 5 Strengths and limitations

A significant strength of our study is the use of multiple causal discovery algorithms, enhancing the robustness and interpretability of the results. This approach marks a departure from traditional approaches that predominantly rely on correlations. This novel approach facilitates a nuanced understanding of the intricate “cause-and-effect” dynamics underlying NSSI behaviors in children and adolescents, particularly in the unique context of the COVID-19 pandemic. By pinpointing fundamental causative elements, our study lays the foundation for more targeted and efficacious interventions that address root causes, providing both immediate and sustained psychological health benefits.

Nevertheless, the study has limitations, notably the influence of variable ordering on model outcomes. We've addressed this by running numerous iterations, lending consistency to our findings despite potential variable ordering effects.

While the adoption of causal algorithms marks an advancement in our analysis, they cannot fully negate the impact of unobserved confounders or bidirectional relationships. The cross-sectional data limits temporal causality claims, necessitating further validation with longitudinal studies.

Additionally, while AI methods enhance efficiency and aid in the application of causal inference to health data, they are not infallible. The necessity for human judgment remains a key component in the interpretative process, not least because different AI algorithms might yield varying interpretations or conclusions (55). This involves using different AI algorithms for mutual validation and conducting repeated experiments to test their robustness. This dual approach of leveraging both AI and human judgment facilitates a more nuanced and robust analysis than could be achieved by either one alone.

In terms of academic recommendations and future directions, there is a compelling need for further exploration of the identified mechanisms underpinning NSSI behaviors. Using longitudinal datasets with time series information, in conjunction with computational causal discovery, can offer more robust and definitive insights into causality. forging interdisciplinary collaborations that meld psychological, societal, and technological insights could provide a more holistic understanding and usher in innovative intervention strategies tailored to the multifaceted challenges of the modern era.

## 6 Conclusions

Drawing on unique computational causal discovery and machine learning methods, this study illuminated the intricate causal network of factors influencing NSSI in children during the COVID-19 pandemic. Our findings underscore nine critical variables intricately interwoven, reflecting the profound effects of the pandemic, academic stress, family dynamics, and individual psychological attributes. The study's insights offer a fresh perspective for devising impactful interventions, emphasizing the significance of addressing root causes, particularly in the wake of unprecedented global challenges.

## Data availability statement

The data analyzed in this study is subject to the following licenses/restrictions: the dataset can only be used for non-commercial, academic research purposes. Requests to access these datasets should be directed to Rui Hu, hu2857911896@163.com.

## Ethics statement

The studies involving humans were approved by Ethics Committee of Sichuan University (Approval No. K2020025). The studies were conducted in accordance with the local legislation and institutional requirements. Written informed consent for participation in this study was provided by the participants' legal guardians/next of kin.

## Author contributions

XG: Conceptualization, Data curation, Investigation, Writing – original draft. LW: Formal analysis, Methodology, Writing – original draft. ZF: Writing – review & editing. ZL: Data curation, Formal analysis, Visualization, Writing – original draft. LL: Supervision, Writing – review & editing. LJ: Supervision, Writing – review & editing. LZ: Funding acquisition, Supervision, Writing – review & editing.
