# Supplementary material for: Factors and pathways of non-suicidal self-injury in children: insights from computational causal analysis
Source: Front Public Health. 2024 Mar 12;12:1305746. doi: 10.3389/fpubh.2024.1305746 (PMC10963487; doi:10.3389/fpubh.2024.1305746)
Supplement: Supplementary file 1 [file Data_Sheet_1.pdf]

Supplementary Material

1 SUPPLEMENTARY FIGURE

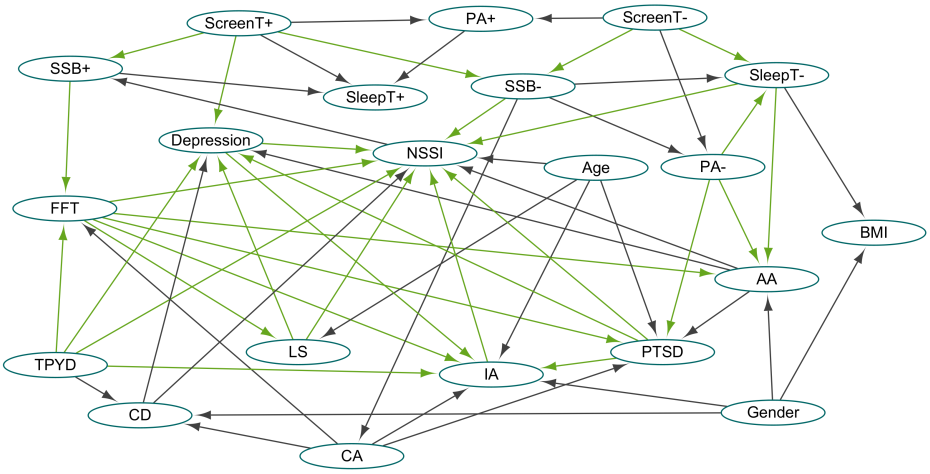

Figure S1. DAG of all NSSI variables - PC Algorithm

2 SUPPLEMENTARY MATERIAL-EXPLANATORY VARIABLES

Sociodemographic Characteristics

1.Gender:

Students self-reported their gender in the questionnaire.

2.Grade:

Students self-reported their grade in the questionnaire.

3.BMI:

BMI values were calculated based on the height and weight data obtained during students' physical examinations. Classification was carried out according to the Chinese BMI screening thresholds for overweight and obesity among school-age children and adolescents aged 6-18, with the categories defined as follows: Normal = 0, Overweight = 1, Obesity = 2.

Mental Health Status

1.Depression:

Depression symptoms were measured using the Epidemiologic Studies Depression Scale (CES-D)(Radloff, 1977), a tool consisting of 20 items that required students to assess the frequency of experiencing depression-related symptoms or feelings over the past week. Having clinical depression risk (total score ≥ 15) = 1, Normal (total score < 15) = 0.

2.Anxiety:

Anxiety was measured using the Screen for Child Anxiety Related Emotional Disorders (SCARED)(Birmaher et al., 1999) Instrument, which employs a 3-point Likert scale and includes 41 items to assess anxiety-related symptoms in children and adolescents. Specific anxiety disorder (total score ≥ 30) = 1, Normal (total score < 30) = 0.

3.AA:

Academic anxiety was assessed by three questions: 'How worried are you about falling behind in your studies?', 'How nervous are you when the teacher hands out the graded papers?' and 'How nervous are you going to be during the exam?'(Shek et al., 1997).

Psychological characteristics

1.TPYD:

Positive Child Development (PCD)(Lerner et al., 2005) represents a positive psychological perspective that prioritizes the encouragement, nurturing, and development of children's developmental strengths, talents, and potential. PCD characteristics are assessed using the Chinese Positive Youth Development Scale, a self-report instrument consisting of 80 items organized into 15 distinct subscales(Shek et al., 2007)

2.LS:

Life satisfaction is assessed using the Life Satisfaction Scale-25(Diener et al., 1985), and the Chinese version of this scale demonstrates acceptable psychometric properties for overall assessment of individual life quality.

3.FFT:

Family dysfunction was assessed using The Chinese Family Assessment Instrument (C-FAI)(Shek and Ma, 2010), which includes five subscales: mutuality, communication, conflict and harmony, parental concern, and parental control.

Caregiver's Mental Health Status

1.CD:

Caregiver's depression symptoms were measured using the Zung Self-rating Depression Scale (SDS)(Zung, 1965). This scale, based on the diagnostic criteria for depression, inquired about their feelings over the past week. Caregivers used a 4-point Likert scale to rate each item. The severity of depression was defined based on standard scores as follows: No depression (< 53)=0, Mild depression(53-62)=1, Moderate depression(63-72)=2, and Severe depression=3 (> 72)=3.

2.CA:

Caregiver's anxiety symptoms were measured using the Zung Self-Rating Anxiety Scale (SAS)(Tao and Gao, 1994), in accordance with anxiety diagnostic criteria. This scale consists of 20 items. Standard scores were calculated for each caregiver, and anxiety severity was categorized as follows: no anxiety(¡50)=0, mild anxiety(50-59)=1, moderate anxiety(60-69)=2, and severe anxiety (> 69)=3.

Behavioural factor

IA:

Internet addiction was measured using the Young Internet Addiction Test (IAT-20)(Young and De Abreu, 2010). This questionnaire consists of 20 items, with each item rated on a scale from 1 to 5, where 1 indicates "rarely" and 5 indicates "always." The categorization is as follows: "Average" online users (total

score 20-49) = 0, Occasional to frequent issues related to internet use (total score 50-79) = 1, Significant issues related to internet use (total score 80-100) = 2

Covid-19-Related Factors

1.PTSD:

Covid-19-related post-traumatic stress disorder (PTSD) was measured using the Children's Impact of Event Scale (13) (CRIES-13)(Lau et al., 2013). This scale comprises 13 items designed to assess the frequency of intrusion, avoidance, and hyperarousal symptoms associated with trauma. Experiencing PTSD symptoms (total score $\geq$ 17) = 1, No PTSD symptoms (total score $<$ 17) = 0.

Covid-19-Related Behavioral Factors

In our study, we also focused on changes in students' behaviors, such as self-reported sleep duration (SleepT±), physical activity time(Zhao et al., 2018) (PA±), screen time (ScreenT±), and consumption of sugary beverages(Organization, 2015) (SSB±), before and during the COVID-19 pandemic. This analysis was pivotal in delineating the pandemic's impact on students' daily routines and lifestyle choices, crucial for understanding the causal pathways leading to NSSI behaviors. For variables with a '+' suffix (e.g., SleepT+, PA+), an increase was coded as '1' (Increased) and no increase as '0' (No increase). For those with a '-' suffix (e.g., SleepT-, PA-), a decrease was coded as '1' (Decreased) and no decrease as '0' (No decrease). This binary coding method allows for a precise quantitative assessment of behavioral changes, significantly enhancing the interpretability of the causal graph results in our study. It provides a structured and quantifiable approach to evaluate how these lifestyle adjustments during the pandemic potentially influence NSSI behaviors among students.

2.SleepT+:

Changes in students' self-reported sleep duration before (prior to January 23) and during (from January 23 until the resumption of in-person classes) the Covid-19 pandemic.(Increased = 1, No increase = 0)

3.SleepT-:

Changes in students' self-reported sleep duration before (prior to January 23) and during (from January 23 until the resumption of in-person classes) the Covid-19 pandemic. (Decreased = 1, No decrease = 0)

4.PA+:

Changes in students' self-reported time spent in physical activity before (prior to January 23) and during (from January 23 until the resumption of in-person classes) the Covid-19 pandemic. (Increased = 1, No increase = 0)

5.PA-:

Changes in students' self-reported time spent in physical activity before (prior to January 23) and during (from January 23 until the resumption of in-person classes) the Covid-19 pandemic. (Decreased = 1, No decrease = 0)

6.Screen T+:

Changes in students' self-reported time spent at electronic screens (for purposes of both online classes and entertainment) before (prior to January 23) and during (from January 23 until the resumption of in-person classes) the Covid-19 pandemic. (Increased = 1, No increase = 0)

7.Screen T-:

Changes in students' self-reported time spent at electronic screens (for purposes of both online classes and entertainment) before (prior to January 23) and during (from January 23 until the resumption of in-person classes) the Covid-19 pandemic. (Decreased = 1, No decrease = 0)

8.SSB+:

Changes in Student Self-Reported Consumption of Sugary Beverages Before (Prior to January 23) and During (From January 23 until the Resumption of In-Person Classes) the Covid-19 Pandemic. (Increased = 1, No increase = 0)

9.SSB-:

Changes in Student Self-Reported Consumption of Sugary Beverages Before (Prior to January 23) and During (From January 23 until the Resumption of In-Person Classes) the Covid-19 Pandemic. (Decreased = 1, No decrease = 0)

Table S1. Names and Measurement for 21 Variables

Variable Abbreviation	Variable Full Name	Measure
NSSI	Non-Suicidal Self-Injury	Nominal
Gender	Students self-reported gender	Nominal
Grade	Students self-reported grade	Ordinal
BMI	Body Mass Index	Ordinal
Depression	Child Depression symptom	Nominal
Anxiety	Child Anxiety Related Emotional Disorder	Nominal
AA	Academic anxiety	Continuous
PTSD	Covid-19-related Post-traumatic Stress Disorder	Nominal
TPYD	Positive Child Development	Continuous
LS	Life Satisfaction	Continuous
FFT	Family dysfunction	Continuous
CD	Caregiver's Depression symptoms	Ordinal
CA	Caregiver's Anxiety symptoms	Ordinal
IA	Internet Addiction	Ordinal
Sleep time+	Increase in students' self-reported sleep duration before and during the Covid-19 pandemic.	Nominal
Sleep time-	Decrease in students' self-reported sleep duration before and during the Covid-19 pandemic.	Nominal
Physical activity+	Increase in students' self-reported time spent in physical activity before and during the Covid-19 pandemic.	Nominal
Physical activity-	Decrease in students' self-reported time spent in physical activity before and during the Covid-19 pandemic.	Nominal
Screen time+	Increase in students' self-reported time spent at electronic screens before and during the Covid-19 pandemic.	Nominal
Screen time-	Decrease in students' self-reported time spent at electronic screens before and during the Covid-19 pandemic.	Nominal
Sugared beverages+	Increase in Student Self-Reported Consumption of Sugary Beverages before and during the Covid-19 pandemic.	Nominal
Sugared beverages-	Decrease in Student Self-Reported Consumption of Sugary Beverages before and during the Covid-19 pandemic.	Nominal

REFERENCES

Birmaher, B., Brent, D. A., Chiappetta, L., Bridge, J., Monga, S., and Baugher, M. (1999). Psychometric properties of the screen for child anxiety related emotional disorders (scared): a replication study. *Journal of the American academy of child & adolescent psychiatry* 38, 1230–1236

Diener, E., Emmons, R. A., Larsen, R. J., and Griffin, S. (1985). The satisfaction with life scale. *Journal of personality assessment* 49, 71–75

Lau, J. T., Yeung, N. C., Yu, X.-n., Zhang, J., Mak, W. W., and Lui, W. W. (2013). Validation of the chinese version of the children's revised impact of event scale (cries) among chinese adolescents in the aftermath of the sichuan earthquake in 2008. *Comprehensive psychiatry* 54, 83–90

Lerner, R. M., Lerner, J. V., Almerigi, J. B., Theokas, C., Phelps, E., Gestsdottir, S., et al. (2005). Positive youth development, participation in community youth development programs, and community contributions of fifth-grade adolescents: Findings from the first wave of the 4-h study of positive youth development. *The journal of early adolescence* 25, 17–71

Organization, W. H. (2015). *Guideline: sugars intake for adults and children* (World Health Organization)

Radloff, L. S. (1977). The ces-d scale: A self-report depression scale for research in the general population. *Applied psychological measurement* 1, 385–401

Shek, D. T., Chan, L., and Lee, T. (1997). Parenting styles, parent-adolescent conflict, and psychological well-being of adolescents with low academic achievement in hong kong. *International Journal of Adolescent Medicine and Health* 9, 233–248

Shek, D. T. and Ma, C. M. (2010). The chinese family assessment instrument (c-fai) hierarchical confirmatory factor analyses and factorial invariance. *Research on Social Work Practice* 20, 112–123

Shek, D. T., Siu, A. M., and Lee, T. Y. (2007). The chinese positive youth development scale: A validation study. *Research on social work practice* 17, 380–391

Tao, M. and Gao, J. (1994). Reliability and validity of zung's self-rating anxiety scale (sas). *Chin J Nerv Ment Dis* 5, 301–3

Young, K. S. and De Abreu, C. N. (2010). *Internet addiction: A handbook and guide to evaluation and treatment* (John Wiley & Sons)

Zhao, L., Wang, Z., Qin, Z., Leslie, E., He, J., Xiong, Y., et al. (2018). Test-retest reliability of physical activity neighborhood environment scale among urban men and women in nanjing, china. *Public Health* 156, 1–7

Zung, W. W. (1965). A self-rating depression scale. *Archives of general psychiatry* 12, 63–70
